# Clinical outcomes of uni-portal non-coaxial spinal endoscopic surgery for severe caudally migrated lumbar disc herniation with free fragments

**DOI:** 10.3389/fsurg.2026.1937822

**Published:** 2026-09-28

**Authors:** Hongshun Zhao, Shihao Zhou, Tianluo Guo, Jianpeng Zheng, Ying Wei, Chuanhai Lin, Liuyue Sunxin, Wenyu Niu, Junhao Sun, Zhihua Xu, En Song, Jiancuo A

**Affiliations:** 1 Department of Spine Surgery, Qinghai Red Cross Hospital, Xining, Qinghai Province, China; 2 Graduate School of Qinghai University, Xining, Qinghai Province, China; 3 Department of Orthopedic Surgery, Datong County People’s Hospital, Datong, Qinghai Province, China; 4 Department of Sports Medicine, The First Affiliated Hospital of Kunming Medical University, Kunming, Yunnan Province, China

**Keywords:** caudally migrated disc herniation, endoscopic lumbar discectomy, lumbar disc herniation, sequestered disc fragment, uni-portal non-coaxial spinal endoscopic surgery

## Abstract

**Objective:**

To evaluate the clinical efficacy and perioperative safety of uni-portal non-coaxial spinal endoscopic surgery (UNSES) for the treatment of severe caudally migrated lumbar disc herniation with free fragments.

**Methods:**

A retrospective study was conducted on 75 consecutive patients with single-level severe caudally migrated lumbar disc herniation who underwent UNSES at a single center between July 2024 and February 2025. Perioperative data and postoperative complications were retrospectively collected. Clinical outcomes were assessed using the visual analogue scale (VAS) for back and leg pain, the Oswestry Disability Index (ODI), and the Japanese Orthopaedic Association (JOA) score before surgery and at 3 days, 3 months, 6 months, and 12 months after surgery. Overall clinical outcomes at the final follow-up were evaluated according to the modified Macnab criteria. Outcome measures at each follow-up time point were compared with baseline values.

**Results:**

All patients successfully underwent UNSES without requiring conversion to open surgery. The mean operative time was 83.81 ± 10.95 min. All incisions healed by primary intention, and no symptomatic epidural hematomas were observed. Perioperative complications included two dural tears and six cases of transient neurological deficits. All patients recovered gradually following conservative management during follow-up. Recurrence occurred in one patient. All patients completed the 12-month follow-up. At the final follow-up, the mean leg pain VAS score decreased from 7.35 ± 0.73 to 1.44 ± 0.55, while the mean back pain VAS score decreased from 6.73 ± 0.83 to 1.37 ± 0.49. The mean ODI decreased from 65.20 ± 6.04 to 14.76 ± 1.78, whereas the mean JOA score increased from 11.96 ± 1.60 to 24.31 ± 1.92. Significant improvements in VAS, ODI, and JOA scores were observed at all postoperative follow-up time points compared with baseline. At the final follow-up, the excellent and good rate according to the modified Macnab criteria was 91%.

**Conclusion:**

UNSES effectively relieves pain and improves lumbar function in patients with severe caudally migrated lumbar disc herniation with free fragments while maintaining an acceptable perioperative safety profile. Short- and mid-term follow-up results suggest that UNSES is a safe and feasible minimally invasive treatment for this challenging type of lumbar disc herniation. However, high-quality prospective studies with longer follow-up are required to further validate its long-term efficacy and potential advantages over other endoscopic techniques.

## 1 Introduction

Lumbar disc herniation (LDH) is a common degenerative spinal disorder and a major cause of low back pain, radicular leg pain, and functional impairment (1). Migrated free-fragment LDH is characterized by complete separation of the herniated nucleus pulposus from the parent intervertebral disc. The detached fragment may migrate cranially or caudally within the spinal canal, leading to nerve root compression and inflammatory irritation. Patients commonly present with persistent low back pain, radiating leg pain, numbness, and sensory or motor deficits (2). Compared with conventional LDH, severe caudally migrated free-fragment LDH is more surgically challenging because the lesion is deeper and the disc fragment migrates farther. Herniated fragments are frequently located beneath the pedicle, within the lateral recess, or along the ventral aspect of the spinal canal. These locations make surgical access difficult and increase the technical complexity of adequate exposure and complete fragment removal. For patients who do not respond to standardized conservative treatment, surgical decompression remains an important option for relieving neural compression and restoring neurological function. Conventional open surgery provides a wide operative field and reliable decompression. However, it is associated with greater disruption of the paraspinal muscles, posterior bony structures, and ligamentous complex. This disruption may increase postoperative pain, delay functional recovery, and compromise spinal stability (3, 4). In recent years, percutaneous endoscopic lumbar discectomy (PELD) has been widely used for LDH because it is minimally invasive and is associated with limited soft-tissue injury and rapid postoperative recovery. Nevertheless, PELD remains limited in patients with severe caudally migrated LDH. When the migrated fragment deviates from the axis of the working channel, the linear endoscopic trajectory may restrict access to the target lesion. This limitation may increase the risk of incomplete fragment removal and inadequate neural decompression (5). In addition, anatomical factors, including a high iliac crest, foraminal stenosis, ligamentum flavum hypertrophy, and lateral recess stenosis, may hinder working-channel placement and limit instrument maneuverability. These factors may increase nerve root traction and require repeated adjustment of the surgical trajectory (6, 7). Unilateral biportal endoscopy (UBE) separates the viewing portal from the working portal. This separation improves instrument mobility and expands the operative field within the spinal canal. UBE has shown advantages in the treatment of lateral recess stenosis and complex LDH (8). However, UBE requires a high level of technical proficiency, including coordinated bimanual manipulation, stable instrument control, clear visualization under continuous irrigation, and effective hemostasis. Therefore, the learning curve is relatively steep (9). Furthermore, in patients with severe spinal canal stenosis, dual-portal manipulation may increase instrument interference, restrict the working space around the nerve root, and introduce irrigation pressure–related risks. Consequently, clinical outcomes and safety may be influenced by surgeon experience and perioperative management (10).

Uni-portal non-coaxial spinal endoscopic surgery (UNSES) is an emerging endoscopic technique for the minimally invasive treatment of spinal disorders (11–13). Unlike conventional coaxial endoscopic surgery, UNSES uses a non-coaxial approach through a single skin incision. This approach separates the endoscopic viewing axis from the trajectory of the surgical instruments. This design improves instrument maneuverability and expands the working space while minimizing soft-tissue disruption. In addition, UNSES can accommodate various conventional surgical instruments and allows dynamic adjustment of the viewing and working angles according to lesion location. These features may improve access to caudally migrated disc fragments, facilitate their removal, and reduce the need for repeated cannula repositioning or extensive bony resection. To date, evidence on the use of UNSES for severe caudally migrated free-fragment LDH remains limited. Its clinical efficacy and safety have not been systematically evaluated. Therefore, this retrospective study analyzed the clinical outcomes of patients with single-level severe caudally migrated free-fragment LDH treated with UNSES. The aim was to evaluate the short- to mid-term clinical efficacy and perioperative safety of this technique and to provide evidence for selecting minimally invasive surgical strategies for this challenging subtype of LDH.

## 2 Materials and methods

This was a single-center retrospective study. A total of 75 patients with single-level severe caudally migrated free-fragment lumbar disc herniation were included. All patients underwent UNSES at Qinghai Red Cross Hospital between July 2024 and February 2025. Endoscopic removal of the herniated nucleus pulposus and neural decompression was performed using UNSES. Demographic and perioperative data were collected. These included age, sex, height, weight, body mass index (BMI), responsible spinal level, American Society of Anesthesiologists (ASA) classification, operative time, and intraoperative and postoperative complications. All procedures were performed by a single senior spine surgeon with extensive experience in minimally invasive lumbar surgery. The study was approved by the Ethics Committee of Qinghai Red Cross Hospital (Approval No. KY-2024-60) and was conducted in accordance with the principles of the Declaration of Helsinki. Inclusion criteria: (1) patients presented with low back pain, radiating leg pain, numbness, or sensory and motor deficits. Neurological signs were clearly identified, and lumbar CT and MRI findings were consistent with clinical symptoms; (2) preoperative MRI demonstrated single-level lumbar disc herniation with a free disc fragment that had lost continuity with the parent intervertebral disc and migrated caudally. The extent of migration was assessed on sagittal MRI using the intervertebral disc level and adjacent pedicle landmarks as anatomical references for zonal classification. Migration into the far-down region (Zone 4) was defined as severe caudal migration. Only patients with free-fragment lumbar disc herniation meeting this radiological criterion were included; (3) symptoms persisted after at least 3 months of standardized conservative treatment, or patients experienced severe and intolerable pain with poor response to conservative management; (4) patients underwent UNSES and had complete clinical and follow-up data available. Exclusion criteria; (1) mild-to-moderate caudally migrated, cranially migrated, or non-migrated disc herniation were excluded; (2) multilevel disc herniation or an unclear responsible segment were excluded; (3) recurrent lumbar disc herniation or prior lumbar spine surgery were excluded; (4) foraminal disc herniation, severe central canal stenosis, severe foraminal stenosis, or other spinal conditions requiring simultaneous surgical treatment were excluded; (5) calcified disc herniation or cases in which bony compression was the primary compressive factor were excluded; (6) segmental instability, lumbar spondylolisthesis, infection, tumor, or fracture at the responsible level were excluded; (7) cauda equina syndrome or cases requiring emergency open decompression were excluded; (8) patients with severe cardiopulmonary, hepatic, or renal dysfunction and poor surgical tolerance were excluded; (9) patients with incomplete follow-up or missing imaging data were excluded.

### 2.1 Surgical technique

All procedures were performed under general anesthesia. After induction of anesthesia, the patients were placed in the prone position. Soft pads were placed beneath the chest and pelvis to allow the abdomen to hang freely. The lumbar spine was slightly flexed to widen the interlaminar space. After routine skin preparation and sterile draping, the target level was identified using anteroposterior and lateral C-arm fluoroscopy. The midline of the spinous processes and the target intervertebral disc level were marked on the skin. A longitudinal incision was made approximately 1.0–1.5 cm lateral to the midline at the target level, serving as the single skin entry point for UNSES. A 30° endoscope with an outer diameter of 4.0 mm and a 12 mm-outer-diameter working cannula were used. The endoscope and surgical instruments were introduced through the same skin entry point. A non-coaxial working space was established by dynamically adjusting the relative orientation of the endoscopic viewing axis and instrument trajectory. This configuration facilitated access to the caudally migrated free fragments and provided greater freedom for instrument manipulation. After incision of the skin and subcutaneous tissue, blunt dissection was performed toward the interlaminar space, and the surgical corridor was established under fluoroscopic guidance. The working cannula and endoscope were then introduced, and the inferior laminar margin, interlaminar space, and ligamentum flavum at the target level were exposed. Soft tissues surrounding the interlaminar space were cleared according to the location of the caudally migrated fragment and the extent of nerve root compression on preoperative imaging. When osseous structures limited lesion exposure or instrument manipulation, a 4.0 mm drill bit was used for limited resection of the laminar edge or medial facet. The extent of bone removal was minimized to preserve the posterior bony structures and facet joint integrity. Under direct endoscopic visualization, the ligamentum flavum was incised or partially resected to expose the dural sac and compressed nerve root. The endoscopic viewing angle and instrument trajectory were dynamically adjusted to explore the shoulder and axillary regions of the nerve root, lateral recess, and ventral spinal canal. After the dural sac, nerve root, and free disc fragment were clearly identified, adhesions between the fragment and surrounding tissues were gently released using a nerve dissector. Excessive traction on the nerve root and dural sac was avoided. For deeply migrated fragments, exploration was extended caudally along the course of the nerve root. The free fragments were then removed piecemeal with pituitary forceps under direct visualization. For fragments located beneath the pedicle, within the deep lateral recess, or in the ventral spinal canal, the instrument trajectory and endoscopic viewing angle were adjusted to enable targeted removal. Blind probing and excessive bony resection were avoided. After fragment removal, the ventral spinal canal, lateral recess, and caudally migrated region were re-explored to confirm the absence of residual free fragments. Adequate decompression was confirmed by relaxation and restoration of pulsation of the nerve root, together with complete relief of dural sac compression. Hemostasis was achieved using a radiofrequency electrode, and the surgical field was thoroughly irrigated. After confirming the absence of active bleeding, the endoscope and working cannula were removed. The incision was closed in layers and covered with a sterile dressing (Figure 1).

**Figure 1:**
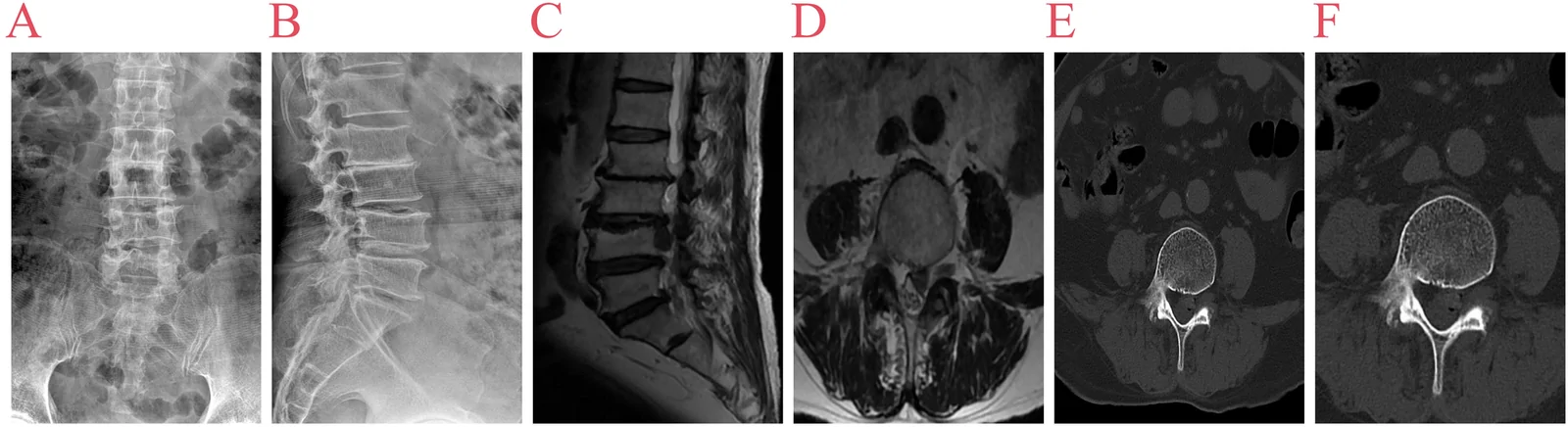
**(A)** Preoperative anteroposterior lumbar spine x-ray; **(B)** preoperative lateral lumbar spine x-ray; **(C)** preoperative sagittal lumbar MRI; **(D)** preoperative axial lumbar MRI; **(E,F)** postoperative axial lumbar CT images showing bony structural changes of the vertebral body and spinal canal.

### 2.2 Statistical analysis

Statistical analyses were performed using SPSS version 27.0. Categorical variables are presented as frequencies and percentages [*n* (%)]. The normality of continuous variables was assessed using the Shapiro–Wilk test. The VAS scores for low back and leg pain, ODI, and JOA scores at all assessment time points were normally distributed and are therefore presented as mean ± standard deviation (mean ± SD). Paired *t*-tests were used to compare preoperative values with those obtained at each postoperative follow-up time point. A *P* value <0.05 was considered statistically significant.

## 3 Results

### 3.1 Patient characteristics

This retrospective study included 75 patients who underwent surgery. The patients' demographic and clinical characteristics are summarized in Table 1. The mean patient age was 50.93 ± 19.91 years, and the cohort consisted of 32 men and 43 women. According to the American Society of Anesthesiologists (ASA) Physical Status Classification System, 37 patients (49%) were classified as ASA class I, 35 (47%) as class II, and 3 (4%) as class III. The affected spinal levels were L3–L4 in 3 patients (4%), L4–L5 in 43 patients (57%), and L5–S1 in 29 patients (39%).

**Table 1:** Patient demographics.

Variables	Total (*n* = 75)
Age, Mean ± SD	50.93 ± 19.91
BMI, Mean ± SD	23.83 ± 2.78
Gender (*n*%)	
Male	32 (43)
Female	43 (57)
Height, Mean ± SD	168.17 ± 8.95
Weight, Mean ± SD	66.51 ± 11.68
ASA classification (*n*%)	
I	37 (49)
II	35 (47)
III	3 (4)
Disc level (*n*%)	
L3–4	3 (4)
L4–5	43 (57)
L5–S1	29 (39)

### 3.2 Clinical outcomes

All 75 patients successfully underwent UNSES, with a mean operative time of 83.81 ± 10.95 min. No patient required conversion to open surgery, and all patients completed the 12-month follow-up. The mean VAS score for low back pain decreased from 6.73 ± 0.83 preoperatively to 3.28 ± 0.74 at 3 days, 2.59 ± 0.72 at 3 months, 2.32 ± 0.55 at 6 months, and 1.37 ± 0.49 at 12 months after surgery. The corresponding mean VAS scores for leg pain decreased from 7.35 ± 0.73 preoperatively to 3.19 ± 0.42, 2.52 ± 0.72, 1.87 ± 0.78, and 1.44 ± 0.55, respectively. The mean ODI decreased from 65.20 ± 6.04 preoperatively to 26.56 ± 3.89, 21.04 ± 4.89, 15.65 ± 2.87, and 14.76 ± 1.78 at the corresponding follow-up time points. The mean JOA score increased from 11.96 ± 1.60 preoperatively to 17.41 ± 1.38, 20.49 ± 1.20, 22.83 ± 1.39, and 24.31 ± 1.92 at the corresponding follow-up time points. Compared with preoperative values, VAS scores for low back and leg pain and ODI scores were significantly lower, whereas JOA scores were significantly higher at all postoperative follow-up time points (all *P* < 0.001; Table 2 and Figure 2). At the final follow-up, outcomes according to the modified MacNab criteria were excellent in 27 patients (36%), good in 41 (55%), fair in 5 (7%), and poor in 2 (3%), resulting in an excellent-to-good rate of 91%. Intraoperative dural tears occurred in 2 patients (3%). Both patients were managed conservatively with bed rest for 1 week. The associated symptoms subsequently resolved, with no persistent neurological deficits. Transient lower-extremity numbness occurred in 6 patients (8%). These patients received neurotrophic medication and rehabilitation therapy, and their symptoms resolved within 3 weeks after surgery. Postoperative recurrence occurred in 1 patient (1%). After reassessment of the clinical symptoms and imaging findings, the patient underwent revision UNSES (Table 3).

**Table 2:** Clinical outcome during follow-up time points.

Variables	Total (*n* = 75)
Lumbar VAS score	
Pre-operation	6.73 ± 0.83
3 days after operation	3.28 ± 0.74
3 months after operation	2.59 ± 0.72
6 months after operation	2.32 ± 0.55
12 months after operation	1.37 ± 0.49
Leg VAS score	
Pre-operation	7.35 ± 0.73
3 days after operation	3.19 ± 0.42
3 months after operation	2.52 ± 0.72
6 months after operation	1.87 ± 0.78
12 months after operation	1.44 ± 0.55
ODI (%)	
Pre-operation	65.20 ± 6.04
3 days after operation	26.56 ± 3.89
3 months after operation	21.04 ± 4.89
6 months after operation	15.65 ± 2.87
12 months after operation	14.76 ± 1.78
JOA score	
Pre-operation	11.96 ± 1.60
3 days after operation	17.41 ± 1.38
3 months after operation	20.49 ± 1.20
6 months after operation	22.83 ± 1.39
12 months after operation	24.31 ± 1.92
Operation time	83.81 ± 10.95

**Figure 2:**
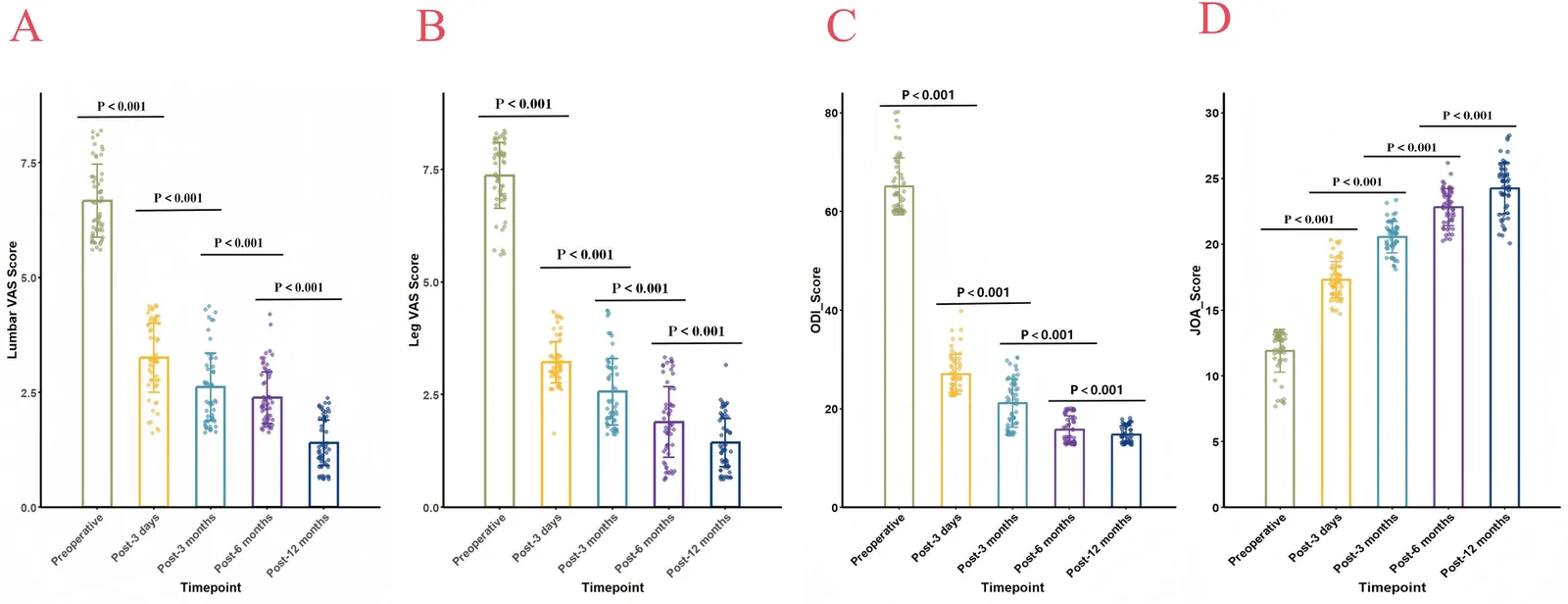
Changes in clinical outcome scores at different time points. **(A)** Low back pain VAS score; **(B)** leg pain VAS score; **(C)** ODI score; **(D)** JOA score.

**Table 3:** Clinical outcomes at the final follow-up and postoperative complications.

Variable	Value
Therapeutic effect	
Excellent	27 (36)
Good	41 (55)
Fair	5 (7)
Poor	2 3)
Excellent/good rate (%)	68 (91)
Complications	
Dural laceration	2 (3)
Transient lower limb numbness	6 (8)
Postoperative recurrence	1 (1)

## 4 Discussion

The treatment of severely caudally migrated sequestrated lumbar disc herniation remains technically challenging because of its distinctive anatomical location and lesion morphology. After penetrating the annulus fibrosus, the sequestrated nucleus pulposus may migrate caudally and separate from the parent disc level. It can extend to the posterior margin of the inferior vertebral body, the subpedicular region, the deep lateral recess, or the ventral aspect of the spinal canal (14). As a result, the lesion may be obscured cranially by the inferior endplate of the cranial vertebral body and the posterior margin of the parent disc. With further caudal migration, it may approach the superior margin of the caudal vertebral body, the medial pedicle wall, and the traversing nerve root. These anatomical constraints create a narrow and irregular working corridor within the spinal canal (4). Unlike conventional lumbar disc herniation, which is usually centered near the intervertebral disc space, caudally migrated sequestrations are displaced inferiorly along the longitudinal axis of the spinal canal. Consequently, the lesion may be markedly misaligned with the trajectory of standard surgical approaches, which makes direct access more difficult (15). In addition, the migrated fragment is surrounded by several osseous structures, including the posteroinferior margin of the cranial vertebral body, the posterosuperior margin of the caudal vertebral body, the medial pedicle wall, the inferior laminar edge, and the medial facet. When the fragment extends into the subpedicular region or behind the caudal vertebral body, it is often concealed by the inferior laminar edge, the ligamentum flavum, and the medial facet. This limits direct endoscopic visualization (16). The close anatomical relationship between the migrated fragment and adjacent neural structures further increases surgical complexity. The dural sac and traversing nerve root are usually located dorsal or lateral to the lesion. Some sequestrated fragments may also be hidden in the axillary region, shoulder region, or ventral aspect of the nerve root. Therefore, fragment removal must be performed with meticulous manipulation near vulnerable neural tissues. In patients with a prolonged disease course, local inflammation, epidural venous engorgement, fibrous adhesions, and ligamentum flavum hypertrophy may further reduce the available working space. These changes can make neural identification, fragment mobilization, and complete excision more difficult (17). In this anatomical context, the main challenge of minimally invasive surgery is not merely to reduce incision size. Rather, it is to establish a stable, accurate, and minimally disruptive surgical corridor despite osseous obstruction, adjacent neural structures, and distal fragment migration (18). For far caudally migrated sequestrations, approaches based on the original disc level or a fixed interlaminar window may create a marked mismatch between the instrument trajectory and the target lesion. As a result, adequate exposure of the posterior margin of the caudal vertebral body, the subpedicular region, and the deep lateral recess may be limited (19). Excessive bone removal to improve visualization may compromise the lamina, facet joint, and ligamentum flavum complex. Conversely, blind exploration toward the caudal spinal canal under limited visualization or inadequate localization may increase the risk of nerve root traction, dural irritation, incidental durotomy, and residual disc fragments (20). Therefore, the key objective of minimally invasive treatment is to create a safe and precise operative pathway to the migrated fragment while minimizing disruption to osseous structures and adjacent neural tissues.

In response to these technical challenges, the main advantage of UNSES is its ability to reconstruct the operative trajectory. Rather than relying on a fixed corridor, UNSES separates the viewing axis from the instrument trajectory through a single skin entry point. The endoscope allows continuous visualization of key anatomical structures, including the dural sac, nerve root, ligamentum flavum, laminar edge, and lateral recess. Surgical instruments can then be angulated according to the direction of fragment migration. This configuration allows the instrument trajectory to be aligned more closely with lesions beneath the pedicle, behind the caudal vertebral body, or along the ventral spinal canal. Direct visualization and neural protection can be maintained throughout the procedure. Therefore, UNSES appears well suited to the anatomical features of severely caudally migrated sequestrated lumbar disc herniation. For sequestrated fragments displaced beyond the original disc level, UNSES allows dynamic adjustment of the endoscopic viewing angle and instrument direction. Multiple exploration and retrieval trajectories can therefore be established through a limited bony window, reducing reliance on a single linear corridor (21). Clinically, adequate exposure can be achieved by modifying the spatial relationship between visualization and instrumentation, rather than by increasing bony resection. This may improve lesion accessibility and surgical precision while reducing neural traction and preserving osseous structures. These features may facilitate targeted decompression and more complete removal of distally migrated fragments. The non-coaxial working space created by UNSES may also improve endoscopic manipulation in anatomically complex regions. For fragments located in the axillary or shoulder region of the nerve root, the deep lateral recess, or the ventral spinal canal, the relationship between the lesion and adjacent neural structures can first be identified under direct endoscopic visualization. Dissection, fragment retrieval, and subsequent inspection can then be performed through relatively safe anatomical planes. This strategy represents a shift from a corridor-oriented approach to a lesion-oriented approach. The operative pathway can be adjusted intraoperatively according to lesion location and surrounding anatomy, rather than being constrained by a predefined trajectory. These lesions are also frequently associated with epidural venous engorgement, tissue adhesions, and varying degrees of degenerative hypertrophy. These factors may compromise endoscopic visualization and interfere with neural identification and complete fragment removal (22, 23). By separating the viewing axis from the instrument trajectory, UNSES may reduce interference between the endoscope and instruments. It may also help maintain a stable and clear operative field under continuous irrigation. This may improve the accuracy and completeness of fragment removal. In the present study, UNSES was successfully completed in all 75 patients without conversion to open surgery. This finding supports the technical feasibility of this approach for severely caudally migrated sequestrated lumbar disc herniation. At the 12-month follow-up, VAS scores for back and leg pain, ODI, and JOA scores had significantly improved compared with baseline. In addition, 91% of patients achieved excellent or good outcomes according to the modified MacNab criteria. These results indicate favorable short- to mid-term outcomes in terms of decompression, pain relief, and functional recovery. However, perioperative complications were observed in several patients. Incidental dural tears occurred in two patients. In severely caudally migrated lesions, the sequestrated fragment may remain adherent to the dural sac for a prolonged period. Local inflammation and fibrous adhesions may obscure the anatomical planes among the fragment, ligamentum flavum, and dura mater. When the lesion is located beneath the pedicle or deep in the ventral spinal canal, the restricted working space increases the difficulty of dissection and fragment retrieval. Meticulous protection of the dural sac is therefore required. Consequently, dural injury may be more likely during the treatment of deeply located lesions. Transient lower-limb numbness was observed in six patients. This symptom may be related to intraoperative nerve root traction, postoperative edema, thermal effects from radiofrequency ablation, or transient reactive changes after decompression. In severely caudally migrated lesions, prolonged compression may cause nerve root congestion, edema, and increased sensitivity. Therefore, transient postoperative sensory disturbances may occur even when adequate decompression is achieved under direct visualization. In most cases, these symptoms gradually resolve after neural compression is relieved and local inflammation subsides. Previous studies have reported postoperative recurrence rates of LDH of 5%–21% (24, 25). Recurrence is generally associated with disc degeneration, annular defects, residual disc material, postoperative mechanical loading, and individual biomechanical factors. In the present study, recurrent herniation occurred in only one patient, corresponding to a recurrence rate of 1.3%. This rate was lower than those reported in previous studies. This finding suggests that UNSES may facilitate thorough removal of migrated disc fragments in this lesion type. However, residual fragments or recurrent herniation remain possible because these lesions often have long migration distances, concealed locations, local adhesions, and osseous obstruction. The completeness of fragment removal may also depend on the surgeon's ability to recognize endoscopic anatomy, control non-coaxial instruments, and adequately re-explore the caudally migrated region. Several limitations should be acknowledged. First, this was a single-center retrospective study with a relatively small sample size. Moreover, all procedures were performed by a single surgeon with extensive experience in minimally invasive lumbar surgery. Therefore, selection bias and surgeon-related effects cannot be excluded, and the generalizability of the findings may be limited. Second, the 12-month follow-up primarily reflects the short- to mid-term outcomes of UNSES for severe caudally migrated lumbar disc herniation with free fragments. Longer-term follow-up is needed to evaluate recurrence, changes in segmental stability, and sustained functional recovery. Third, no comparison group undergoing PELD, UBE, or open surgery was included. Consequently, the potential advantages of UNSES over other surgical techniques in terms of lesion accessibility, adequacy of decompression, and complication control cannot be directly determined. Fourth, outcomes were primarily evaluated using clinical scores and the modified MacNab criteria. Quantitative preoperative and postoperative radiological parameters were not systematically assessed, including the extent of migrated fragment removal, spinal canal decompression area, extent of bony resection, intervertebral disc height, segmental stability, and lumbar sagittal parameters. Therefore, the adequacy of decompression and preservation of spinal structures could not be fully evaluated. Finally, the outcomes of UNSES may depend on the surgeon's ability to identify endoscopic anatomy, manipulate non-coaxial instruments, and overcome the associated learning curve. However, the learning curve was not evaluated in the present study. Future large-scale, multicenter, prospective comparative studies are warranted. These studies should incorporate standardized radiological and functional assessments with long-term follow-up to further define the indications, safety profile, and long-term clinical value of UNSES for severe caudally migrated lumbar disc herniation with free fragments.

## 5 Conclusion

UNSES effectively relieves pain and improves lumbar function in patients with severe caudally migrated lumbar disc herniation with free fragments while maintaining an acceptable perioperative safety profile. Short- and mid-term follow-up results suggest that UNSES is a safe and feasible minimally invasive treatment for this challenging type of lumbar disc herniation. However, high-quality prospective studies with longer follow-up are required to further validate its long-term efficacy and potential advantages over other endoscopic techniques.

## Data Availability

The raw data supporting the conclusions of this article will be made available by the authors, without undue reservation.
